# Variability in transmissibility of the 2009 H1N1 pandemic in Canadian communities

**DOI:** 10.1186/1756-0500-4-537

**Published:** 2011-12-13

**Authors:** Luiz C Mostaço-Guidolin, Amy Greer, Beate Sander, Jianhong Wu, Seyed M Moghadas

**Affiliations:** 1 Centre for Disease Modelling, York University, 4700 Keele Street, Toronto, ON, M3J 1P3, Canada; 2 Modeling and Projection Section, Professional Guidelines and Public Health Practice Division, Centre for Communicable Diseases and Infection Control, Public Health Agency of Canada, 180 Queen Street W., Toronto, ON, M5V 3L7, Canada; 3 Division of Epidemiology, Dalla Lana School of Public Health, University of Toronto, 155 College Street, Toronto, ON, M5T 3M7, Canada; 4 Public Health Ontario, 480 University Avenue, Toronto, ON, M5G 1V2, Canada

## Abstract

**Background:**

The prevalence and severity of the 2009 H1N1 pandemic appeared to vary significantly across populations and geographic regions. We sought to investigate the variability in transmissibility of H1N1 pandemic in different health regions (including urban centres and remote, isolated communities) in the province of Manitoba, Canada.

**Methods:**

The Richards model was used to fit to the daily number of laboratory-confirmed cases and estimate transmissibility (referred to as the basic reproduction number, *R*_0_), doubling times, and turning points of outbreaks in both spring and fall waves of the H1N1 pandemic in several health regions.

**Results:**

We observed considerable variation in *R*_0 _estimates ranging from 1.55 to 2.24, with confidence intervals ranging from 1.45 to 2.88, for an average generation time of 2.9 days, and shorter doubling times in some remote and isolated communities compared to urban centres, suggesting a more rapid spread of disease in these communities during the first wave. For the second wave, *R_e_*, the effective reproduction number, is estimated to be lower for remote and isolated communities; however, outbreaks appear to have been driven by somewhat higher transmissibility in urban centres.

**Conclusions:**

There was considerable geographic variation in transmissibility of the 2009 pandemic outbreaks. While highlighting the importance of estimating *R*_0 _for informing health responses, the findings indicate that projecting the transmissibility for large-scale epidemics may not faithfully characterize the early spread of disease in remote and isolated communities.

## Background

Although the overall Canadian experience of the 2009 H1N1 pandemic influenza was perceived to be relatively mild for most individuals, the disease disproportionately affected several vulnerable and underserved communities, including First Nation reserves in northern Manitoba, remote and isolated communities in Nunavut, and Aboriginal communities on Vancouver Island [1-3]. The variability in disease outcomes is often described by factors pertinent to the population demographics, environmental characteristics, underlying health conditions, and healthcare access [3-6]. A direct effect of these factors appears on the transmissibility of disease in different population settings, which may contribute to larger outbreaks in vulnerable communities as a result of differential prevalence [7].

Transmissibility can be described as the basic reproduction number (*R*_0_), which in the epidemiological context, is defined as the average number of new cases generated by an infectious individual in a fully susceptible population [8]. This quantity, once estimated for a new disease, can be used to identify target strategies for mitigating disease outcomes. Following the emergence of the H1N1 pandemic strain, attempts were made to estimate *R*_0_, largely for urban population settings [9-12]. These estimates suggest that the novel H1N1 strain was less transmissible (range: 1.2-1.7) than the 1918-1919 pandemic virus (range: 1.8-3.0) [12]. However, H1N1 virus transmission appeared to vary significantly in remote and isolated communities, and among vulnerable population groups [1,3], and therefore prior estimates of *R*_0 _for the urban population settings may not be applicable to devising targeted measures for future planning in these communities.

Given the experience of the 2009 pandemic in the province of Manitoba, Canada, with severe outbreaks in different health regions during both spring and fall waves, we sought to investigate the variability in transmissibility of the disease using the data for laboratory confirmed cases of H1N1 infection. The Richards model [13] was applied and fit to cumulative incidence of confirmed cases to estimate the transmissibility and turning points of the outbreaks in different population settings, and compared the results for the first and second waves. This modelling approach allows us to predict changes in the course of a single outbreak, determine the cumulative incidence at the turning point of the epidemic, and project the final number of identified cases (the cumulative incidence of infection) at the end of epidemic. In practical terms, determining the turning point of an outbreak will provide important public health information for timely implementation of effective intervention strategies. The results are compared with those of previous studies on transmissibility of pandemic H1N1 influenza. Finally, the findings are placed in the context of public health practice for future planning regarding the prevention and control of emerging infectious diseases, and the impact of constraints imposed by data and the model on the results presented in this study are discussed.

## Materials and methods

To estimate plausible ranges for *R*_0_, the Richards model [13] was employed to fit to data collected for laboratory-confirmed cases of H1N1 infection in a single outbreak. This type of modelling, which describes the dynamics of cumulative incidence of infection, has been widely used in the study of plant disease epidemics [14,15], and more recently for the epidemics in human populations [16-19].

### The model

When *I(t) *is used to represent the cumulative number of confirmed cases of pandemic H1N1 infection on day *t *during the outbreak, the Richards model (the rate of change in *I *with time) can be expressed as

(1) $$I^{'}=rI\left[1-{\left(\frac{I}{K}\right)}^{a}\right],$$

where *r *represents the intrinsic growth rate of *I *in the absence of any limitation to disease spread; *K *is the final size (maximum cumulative cases) of *I*; and *a *measures the extent of deviation from the S-shaped dynamics of the classical logistic growth model [20]. This model describes the epidemic dynamics in two phases of fast and slow infection spread with a transition point (turning point), at which the maximum rate of disease incidence occurs. In the slow phase of infection spread (after the turning point), the epidemic peaks and subsequently declines, and therefore the cumulative number of cases eventually saturates at the final size *K*. This effectively means that the Richards model represents the incidence curve of a single wave of infection which consists of a single peak of high incidence with a single turning point for the outbreak. The readers may consult published literature for more details on the Richards model and its application to disease epidemics [17-19,21]. The reproduction number of disease is estimated by determining the rate of disease propagation at the early stages of the epidemic before the turning point. For estimating parameters through model fitting, the explicit solution of the Richards model, given by

(2) $$I\left(t\right)=\frac{K}{{\left[1+e^{-ra\left(t-\tau \right)}\right]}^{\frac{1}{a}}},$$

was used, where *τ *is constant of integration related to the time for change in the growth rate of the cumulative cases. By fitting this solution to the cumulative case counts, the rate of infection spread (*r*) at the onset of each outbreak was obtained. Assuming that the generation time (the average time taken for the primary case to infect secondary cases [22]) follows a gamma distribution with shape parameter *α *and scale parameter *β *[23,24], the reproduction number can be estimated as *R*_0 _= (1 + *rβ*)^*α *^using the moment generation method [25]. The terminology of effective reproduction number, *R_e_*, is used for the second wave, given immunity from the first wave in the population. Turning points of the outbreaks (*t_m_*) were also estimated by determining the inflection points at which *I*(*t*_*m*_) = *K*/(1 + *α*)^1/*α*^. We should point out that previous work [19] has referred to the parameter *τ *as the turning point, which is theoretically incorrect; although in numerical terms it may lead to approximations that are close to the actual turning point. We also estimated the doubling time (the time it takes for the number of cases to double) at the early stages of the outbreak as *t*_*d *_= *T *ln 2(*R*_0 _- 1) [22].

### Generation time

Based on epidemiological studies conducted in the United States for the infector-infectee pairs in households [23], an average generation time of 2.9 days was considered with a standard deviation of 1.4 days for the Gamma distribution with *α *= 4.2 and *β *= 0.68 [24]. We estimated *R*_0 _and *R_e _*using this generation time and also a shorter average generation time of 2.5 days with standard deviation of 0.9 days [26] that follows a Gamma distribution with shape and scale parameters *α *= 7.72 and *β *= 0.324, respectively.

### Data collection

**Figure 1:**
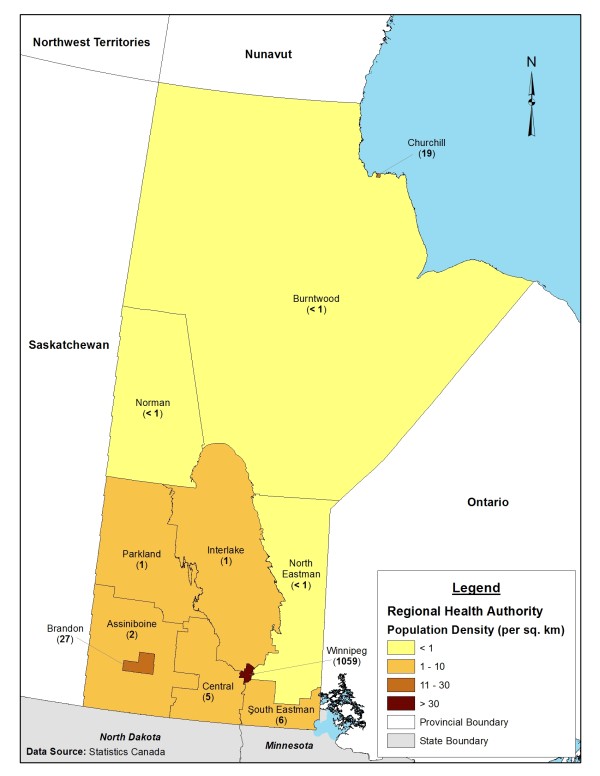
**Manitoba population density by regional health authority**.

Daily number of laboratory-confirmed cases of H1N1 influenza infection were obtained from influenza databases of Manitoba Health for both waves of the 2009 pandemic in spring (total of 891 cases between May 2 and August 5) and fall (total of 1,774 cases between October 1, 2009, and January 3, 2010), classified for each of the 11 health regions in the province of Manitoba, Canada (Figure 1). The number of reported cases per health region (sample size) determined which health regions were included in the first wave analysis (Burntwood, North Eastman, Norman, Interlake, Winnipeg) and second wave analysis (Brandon, Central, Assiniboine, Interlake, Winnipeg). Only health regions with more than 50 identified cases were considered for this study.

A laboratory-confirmed case was defined as an individual with influenza-like illness or severe respiratory illness who tested positive for pandemic H1N1 influenza A virus by real-time reverse-transcriptase PCR (RT-PCR) or viral culture. The first case of H1N1 infection in Manitoba was identified (tested positive) on May 2, 2009, and data were reported by the earliest date of symptom onset, initial care, specimen collection, hospital admission, and/or intensive care unit admission. For the present study, data use was approved by the Human Research Ethics Board of the University of Manitoba (H2009:339) and Health Information Privacy Committee of Manitoba (2009/2010-40).

### Model fitting

The solution of the Richards model was fit to the cumulative number of confirmed cases by applying the nonlinear least squares method using the R-language and environment for statistical computing. All fits were performed starting from the day on which the first laboratory confirmed case was identified in the associated health region. The turning point (*t_m_*) of the outbreaks was determined using the Newton-Raphson method at the inflection point.

## Results

For the average generation time of 2.9 days, estimates of fitting parameters, and *R*_0 _and *R_e _*with 95% confidence intervals for the best fit to data for cumulative reported cases in different health regions are summarized in Tables 1 and 2. Estimates associated with the shorter generation time (2.5 days) are reported in Table 3.

**Table 1:** Estimates of fitting parameters and the basic reproduction number for the first wave of the 2009 pandemic in different health regions in the province of Manitoba

	Parameters			
**Health Region**	***r *(95% CI)**	*K *(CRC)*	***t_m_***	*R*_0 _**(95% CI)**

Burntwood	0.3116 (0.2503-0.4208)	187 (186)	June 5	2.24 (1.93-2.88)

North Eastman	0.1991 (0.1734-0.2390)	56 (56)	June 14	1.70 (1.60-1.88)

Norman	0.1746 (0.1458-0.2217)	57 (58)	June 8	1.60 (1.49-1.80)

Interlake	0.1606 (0.1353-0.2023)	53 (54)	June 14	1.55 (1.45-1.72)

Winnipeg	0.2001 (0.1730-0.2397)	395 (401)	June 9	1.71 (1.60-1.89)

Manitoba	0.1949 (0.1700-0.2306)	878 (891)	June 9	1.69 (1.58-1.84)

*CRC cumulative reported cases. Estimates correspond to the best fit of the Richards model to the cumulative number of confirmed cases. 95% confidence interval for *R*_0 _corresponds to the average generation time of 2.9 days.

**Table 2:** Estimates of fitting parameters and the effective reproduction number for the second wave of the 2009 pandemic in different health regions in the province of Manitoba

	Parameters			
**Health Region**	***r *(95% CI)**	*K *(CRC)*	***t_m_***	*R_e _***(95% CI)**

Brandon	0.2064 (0.1664-0.2787)	97 (98)	Nov 11	1.74 (1.57-2.07)

Central	0.1826 (0.1644-0.2067)	271 (270)	Nov 9	1.63 (1.56-1.74)

Assiniboine	0.1459 (0.1378-0.1553)	201 (202)	Nov 12	1.49 (1.46-1.52)

Interlake	0.1698 (0.1469-0.2049)	149 (148)	Nov 12	1.58 (1.49-1.73)

Winnipeg	0.2470 (0.2127-0.2973)	702(706)	Nov 7	1.92 (1.76-2.17)

Manitoba	0.1899 (0.1731-0.2113)	1764 (1774)	Nov 9	1.67 (1.60-1.76)

*CRC cumulative reported cases. Estimates correspond to the best fit of the Richards model to the cumulative number of confirmed cases. 95% confidence interval for *R_e _*corresponds to the average generation time of 2.9 days.

**Table 3:** Estimates for *R*_0 _and *R_e _*with 95% confidence intervals with the average generation time of 2.5 days

First wave		Second wave	
**Health Region**	*R*_0 _**(95% CI)**	**Health Region**	***R***_***e ***_**(95% CI)**

Burntwood	2.10 (1.83-2.68)	Brandon	1.65 (1.50-1.95)

North Eastman	1.62 (1.52-1.78)	Central	1.56 (1.49-1.65)

Norman	1.53 (1.43-1.71)	Assiniboine	1.43 (1.40-1.46)

Interlake	1.48 (1.39-1.63)	Interlake	1.51 (1.43-1.64)

Winnipeg	1.62 (1.52-1.78)	Winnipeg	1.81 (1.67-2.03)

Manitoba	1.60 (1.51-1.74)	Manitoba	1.59 (1.52-1.67)

### First wave

**Figure 2:**
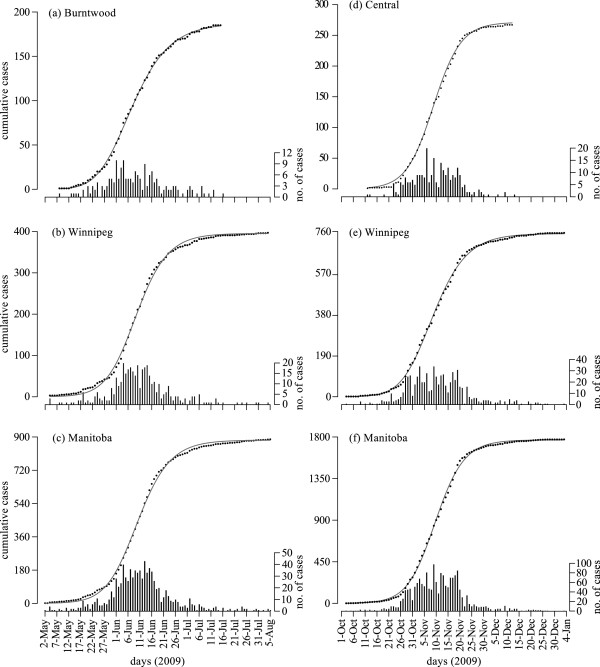
**Model fits (black curves) to the cumulative number of confirmed cases (grey dots) for the first (**a**-**c**) and second (**d**-**f**) waves of pandemic H1N1**. Bar plots represent the daily number of confirmed cases in each region.

The most severely affected health regions during the spring wave were Burntwood, North Eastman, and Norman in northern Manitoba, where the majority of remote and isolated communities, and aboriginal reserves (many of them without road access) are located. A total number of 300 H1N1 cases were confirmed in these regions. Assuming the average generation time of 2.9 days, the mean *R*_0 _was estimated to be 2.24 (95% CI: 1.93-2.88) for Burntwood, 1.70 (95% CI: 1.60-1.88) for North Eastman, and 1.60 (95% CI: 1.49-1.80) for Norman (Table 1). Other impacted health regions included Interlake, and Winnipeg which is the largest urban centre in the province where 57% of the Manitoba population resides. Estimates of *R*_0 _according to the best fit to data for these regions are 1.55 (95% CI: 1.45-1.72) and 1.71 (95% CI: 1.60-1.89), respectively. The variability in these estimates may in part be due to different generation times across populations and geographic areas. When considering a shorter generation time of 2.5 days for Burntwood, estimates of *R*_0 _reduce with a mean value of 2.1 (Table 3), but still remain above those of Winnipeg health region with the average generation time of 2.9 days. Using the data for the entire province, estimates of *R*_0 _and its associated range remain close to those of the Winnipeg health region. Our results indicate that the turning points of epidemics started in the first week of June 2009, with the earliest in Burntwood on June 5 and the latest in North Eastman on June 14. Given the later onset of pandemic outbreaks in Burntwood, the estimated turning points indicate a more rapid spread of disease in northern part of the province. This can be further demonstrated by evaluating the doubling time, which is *t_d _*= 1.6 days for the Burntwood health region with an average generation time of *T *= 2.9 days; while the doubling time for the Winnipeg health region is approximately 1.2 days longer (*t_d _*≈2.8). Best fits for Burntwood, Winnipeg, and the entire province of Manitoba for the spring wave are illustrated in Figure 2a-c.

### Second wave

During the second wave, Central, Brandon, and Assiniboine were the most impacted health regions in the southern part of the province of Manitoba. The mean effective reproduction number *R_e _*was estimated to be 1.63 (95% CI: 1.56-1.74) for Central, 1.74 (95% CI: 1.57-2.07) for Brandon, and 1.49 (95% CI: 1.46-1.52) for Assiniboine health regions with the average generation time of 2.9 days (Table 2). For comparison purposes, *R_e _*was also estimated for Interlake, Winnipeg, and for the entire province. We observed that *R_e _*estimates for Interlake and the entire province remained virtually the same for both waves, but slightly increased for Winnipeg in the second wave. Turning points of the outbreaks were projected between November 7 and 12. The results of parameter estimates are reported in Table 2, and best fits for Central, Winnipeg, and Manitoba are illustrated in Figure 2d-f.

### Two-wave comparison

The occurrence of pandemic outbreaks appeared to be associated with a geographic shift from northern Manitoba (e.g., Burntwood, North Eastman, and Norman) in the first wave to more southern parts of the province (e.g., Assiniboine, Central, and Brandon) in the second wave (Figure 1). Estimates for the first wave indicate that the plausible range of *R*_0 _for the entire province of Manitoba is virtually identical to that obtained for its largest urban centre (the Winnipeg health region). In the second wave, transmissibility appears to have been lower for the entire province than the Winnipeg health region. In comparing timelines of epidemic spread, our estimates of turning points show that the time interval for the pace of faster epidemic growth (the period before turning point) was comparable to that of the first wave, and occurred about 5 to 6 weeks after the onset of outbreaks in Manitoba. This is notwithstanding higher levels of pre-existing immunity and wider use of antiviral drugs in the second wave before vaccine distribution (which were expected to synergistically decelerate the rate of disease spread).

## Discussion and conclusions

In the event of an emerging disease, estimates of transmissibility and other parameters pertinent to the nature of the infection provide critical information for guiding public health in optimizing health policy decisions and mitigating the impact of disease on affected populations [27]. In the case of the 2009 pandemic, estimates of *R*_0 _helped characterize the epidemiological patterns of early disease spread in urban centres with large populations [9,10,12], and adapt preparedness measures to address the urgent situation in these settings [28,29]. However, such estimates were not available for remote and isolated communities and other residential areas with small populations (e.g., Indigenous peoples on reserves), which experienced disproportionate rates of illness with poor outcomes, in particular during outbreaks in the spring wave. In Canada, severe outbreaks were observed in several locations in the province of Manitoba during the first wave, most notably in communities in the Burntwood, Norman and North Eastman health regions.

In contrast to previous work on estimating *R*_0 _[9,10,27], the results of this study suggest a considerable geographic variation in transmissibility of the 2009 pandemic for the first wave, with estimates comparable to those of the 1918 (H1N1-Spanish) pandemic strain between 1.8 and 3.0 [12]. For the second wave in Manitoba, our estimates indicate a reduction in *R_e _*with ranges that are considered to be of moderate transmissibility and comparable to those of the 1957 (H2N2) (range: 1.5-1.7) and 1968 (H3N2) pandemics (range: 1.6-2.2), yet significantly higher than the transmissibility of seasonal influenza (range: 1.1-1.4) [12,30]. The higher estimates of *R*_0 _in the first wave of the 2009 pandemic are linked to outbreaks in remote and isolated communities, and populations of relatively small size; although estimates of the urban centre of the Winnipeg health region are close to the upper bound of previous estimates [12]. Compared to estimates in the province of Ontario (range: 1.25-1.38) [27], and national Canadian estimates (range: 1.12-1.54) [19], the results of this study show that projecting the transmissibility using data for large-scale epidemics may not faithfully characterize the transmission pattern of early spread in rural areas and isolated communities.

The results of this study have several important implications for future planning to meet the threat of novel influenza viruses. Of greatest concern to public health are the geographic spread, severity, time course of an outbreak, and determining the most effective utilization of available social, preventive, and therapeutic resources to mitigate the impacts of an emerging disease. Understanding the relationship between differential severity and differential transmissibility of a disease in distinct populations can inform possible scenarios for allocation and optimal distribution of health resources prior to and during the spread of an infection to reduce vulnerability of the populations and alleviate disease outcomes. Disease management is also concerned with the transmissibility that is closely related to the final size of an epidemic in the long-term disease dynamics. The parameter *R*_0 _has a clear biological significance: it determines how fast the infection will spread through a population that has been previously unexposed to the disease, its meaning is independent of the model used to estimate its value, and it is dimensionless and thus directly comparable across populations and geographic regions. Our investigation in this study provides compelling evidence for the existence of strong links between transmissibility and differential severity of the disease, in particular in communities with limited access to healthcare. In small communities with strong social ties (e.g., Indigenous populations), frequent interactions between individuals are considerably higher than those in large urban centres, which can result in repeated exposure to infection and may account for a higher between-household transmission rate comparable to the rates of secondary household transmission observed at more southern latitudes. Given factors such as multigenerational households, high between-household social interactions, environmental characteristics, limited access to healthcare, and differential prevalence of predisposing health conditions and other types of health disparities, disease control strategies for remote and isolated communities may be significantly different from those applied to urban centres. Early real-time estimates of transmissibility will therefore be crucial for identifying the type and intensity of intervention measures that are effective in disease containment. In the absence of such information during the early stages of the 2009 pandemic, the existing recommendations for the use of antiviral drugs were modified to include the treatment of moderate to severe cases and individuals with pre-existing conditions or those at risk of developing poor outcomes, but to exclude treatment of mild cases or contacts [31]. For future planning, a less conservative strategy may be beneficial for remote and isolated communities that are more likely to suffer from increased rates of disease transmission. A less conservative strategy might include a broader coverage of antiviral drugs with possible extension to prophylaxis of close contacts in these "high-risk" populations. Although not as cost-effective as vaccination, antiviral use for treatment and post-exposure prophylaxis is far more economical than hospitalization or intensive care. Furthermore, without a virus-specific vaccine (which, for example, could have been the case in the fall wave of the H1N1 pandemic had the herald wave not struck in the spring of 2009), antiviral medication may be the only pharmaceutical option and therefore availability and strategic use of drugs is crucial for mitigating disease in these vulnerable communities. With unique population characteristics that place some remote and isolated communities at increased risk for adverse health consequences [1], it is imperative for public health officials to identify transmission characteristics of early spread for the implementation of effective, feasible, and more economical health responses [32,33].

This study has several limitations that warrant further investigation. For estimating the transmissibility of different health regions, we used data for laboratory confirmed cases of H1N1 infection, which may introduce some bias due to differential rates of testing across age groups, over time, and possibly between different health regions. It is difficult to assess the magnitude and direction of such biases, but we understand that they influence estimates and comparative analysis of *R*_0 _within and between health regions. While our results demonstrate the variability in diseases transmissibility in different population settings, identification of factors responsible for such variation remains an important objective for future work, and in the context of the 2009 pandemic, understanding the role of these factors merit further population-specific research. For example, we used estimates of generation times from two previous study, which appear to be higher than the early estimate of 1.9 days for generation times based on the La Gloria data from initial outbreaks in Mexico [9]. These estimates may have been slightly inflated by the inclusion of a small number of imported cases. Nevertheless, for a given generation time, estimates of *R*_0 _for remote and isolated communities will remain higher than those for more urban regions. However, the generation time is a key parameter in our study, and we note that it may vary between different populations with distinct demographic variables, which will in turn affect estimates of the reproduction number. Furthermore, *R*_0 _can be calculated as the product of the frequency of effective contact (leading to transmission) and the average infectious period. It is possible that in remote communities, due to the limited access to health services and medical care, an average infectious individual may experience a longer contagious period than an average infectious person in urban centres, hence contributing to a greater reproduction number without having to make more frequent contact per unit time.

The model employed here does not include the immune status of the populations; yet we understand that estimates of *R*_0 _for the first wave may be affected by pre-existing immunity [34,35], and therefore the actual value of *R*_0 _(when the entire population is susceptible) may be greater than estimates reported here. For the second wave, the effect of pre-existing immunity becomes more pronounced by the reduced level of susceptibility due to infection in the spring wave in northern communities. Furthermore, unlike the first wave, antiviral drugs were used earlier and in a much wider scale [3]. There were also some limitations in data for laboratory confirmed cases of the second wave. For instance, in order to maintain the laboratory capacity and responsiveness, laboratory testing of non-severe cases was suspended on November 20, 2009, unless infected individuals met indications outlined in the Manitoba Health Clinical Care Guidelines for Pandemic H1N1. Although this modification was made well beyond the establishment phase of the second wave, it may have resulted in a significant under-reporting at the peak of pandemic outbreaks, and therefore estimates of the final size of the cumulative number of confirmed cases and other fitting parameters may be influenced. Despite these limitations, the findings of this study underscore the importance of rapid identification of transmission characteristics in the early stages of an emerging disease to identify effective and population specific intervention strategies, and optimize health responses.

## Competing interests

The authors declare that they have no competing interests.

## Authors' contributions

Conceived, designed the study, and collected data: SM. Performed analysis: LMG. Wrote the first draft of the paper: SM. Contributed reagents/materials/analysis tools: AG, BS, JW. All the authors have contributed to the writing of the final version and revision, and approved it.
